# Innovative Approaches to Large-Area Perovskite Solar Cell Fabrication Using Slit Coating

**DOI:** 10.3390/molecules29204976

**Published:** 2024-10-21

**Authors:** Yitong Wang, Zetong Cheng, Junguo Li, Kuanxin Lv, Zhenzhen Li, Hang Zhao

**Affiliations:** College of Metallurgy and Energy, North China University of Science and Technology, Tangshan 063210, China

**Keywords:** slit coating method, large-area perovskites, photoelectric conversion efficiency, preparation process

## Abstract

Perovskite solar cells (PSCs) are gaining prominence in the photovoltaic industry due to their exceptional photoelectric performance and low manufacturing costs, achieving a significant power conversion efficiency of 26.4%, which closely rivals that of silicon solar cells. Despite substantial advancements, the effective area of high-efficiency PSCs is typically limited to about 0.1 cm^2^ in laboratory settings, with efficiency decreasing as the area increases. The limitation poses a major obstacle to commercialization, as large-area, high-quality perovskite films are crucial for commercial applications. This paper reviews current techniques for producing large-area perovskites, focusing on slot-die coating, a method that has attracted attention for its revolutionary potential in PSC manufacturing. Slot-die coating allows for precise control over film thickness and is compatible with roll-to-roll systems, making it suitable for large-scale applications. The paper systematically outlines the characteristics of slot-die coating, along with its advantages and disadvantages in commercial applications, suggests corresponding optimization strategies, and discusses future development directions to enhance the scalability and efficiency of PSCs, paving the way for broader commercial deployment.

## 1. Introduction

Perovskite solar cells (PSCs), evolved from dye-sensitized solar cells (DSSCs), have rapidly emerged as a promising alternative to traditional solar cell technologies [1,2]. Representing the third wave of solar technology advancements, PSCs offer significant advantages over silicon cells, such as lower production costs, tunable bandgap, and higher flexibility [3,4], making them highly suitable for widespread adoption due to their outstanding photoelectric properties. As early as 2009, Kojima A et al. first fabricated perovskite solar cells, achieving an initial efficiency of 3.8% [5]. Over the next decade, significant advancements have propelled the efficiency to over 26% [6], approaching the theoretical limit analyzed by Park [7]. The remarkable advancements in PSC technology have set the stage for exploring large-scale commercialization, yet significant challenges still persist, hindering the full realization of their potential in the solar energy market [8,9]. The decline in power conversion efficiency (PCE) with increased active area hampers scalability and commercial viability. Large-scale deployment relies on maintaining high efficiency across extensive cell areas, which directly affects production scalability and overall cost effectiveness [10]. Additionally, the efficiency and stability of PSCs depend largely on the quality of the perovskite layer, which varies with different synthesis methods [10,11]. Addressing these challenges is essential for unlocking the potential of PSCs in revolutionizing solar energy solutions.

As the pursuit of efficient and scalable production methods for PSCs intensifies, innovative thin-film preparation techniques have played a crucial role in overcoming existing manufacturing challenges. To date, most perovskite solar cells have primarily been developed for small-scale laboratory applications. Transitioning perovskite solar cells from a laboratory environment to large-scale applications requires identifying scalable deposition methods that can maintain high efficiency. Currently, mainstream methods for preparing large-area perovskites include a variety of techniques such as spin coating [12], slit coating, spraying [13], and blade coating [14]. Advanced methods like inkjet printing [15], various evaporation techniques (including vacuum flash evaporation-assisted [16], sequential [17], and vacuum thermal evaporation [18]), chemical vapor deposition [19], the multi-flow gas knife method [20], and drop casting [21] are also employed.

Among these methods, the slit coating method stands out due to its potential to revolutionize the fabrication process of PSCs. Initially developed by Beguin, this method is a continuous process that allows for precise control over film thickness, making it highly compatible with roll-to-roll (R2R) systems [22,23]. The one-step slit coating technique not only ensures high material utilization but also proves to be suitable for large-area and flexible substrate applications, addressing key scalability and cost-effectiveness concerns [24,25,26]. By integrating this advanced coating method, the production of PSCs can achieve new levels of efficiency and adaptability, paving the way for their broader commercial deployment and outstanding photovoltaic performance.

This review provides an overview of the properties of large-area perovskites prepared using slit coating, detailing the implementation mechanism and summarizing its main advantages. It also proposes optimized methods based on existing research and systematically addresses the challenges faced in preparing large-area perovskites with this method, offering insights into future optimization and development directions.

## 2. Scaling up Perovskite Solar Cells: Opportunities and Challenges

**Figure 1:**
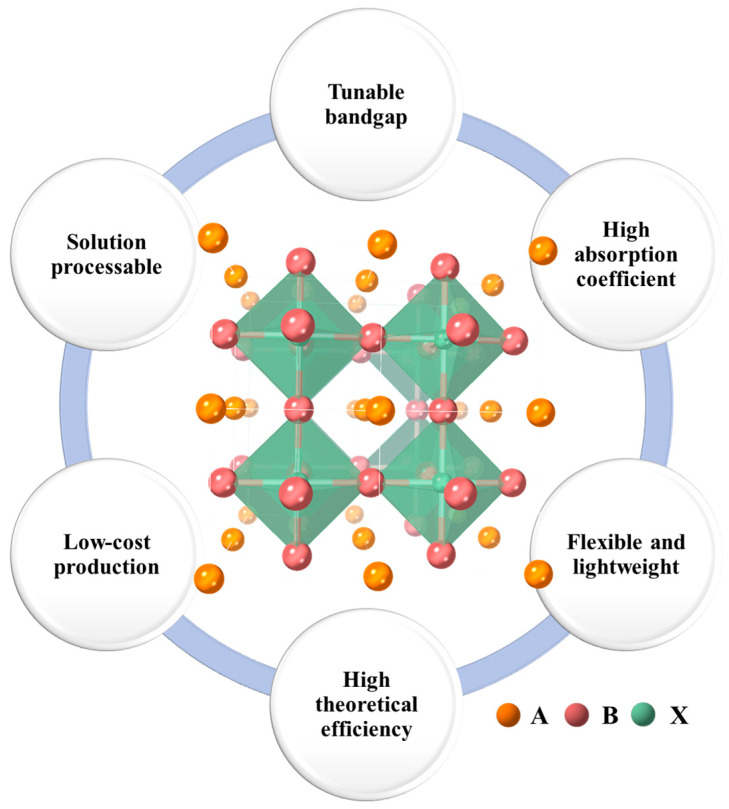
Crystal structure and key advantages of ABX_3_ perovskite materials.

The perovskite structure, typically denoted by the chemical formula ABX_3_, is fundamental to their application in solar cells [27]. In the structure, as illustrated in Figure 1, ‘A’ represents a monovalent cation, which can be an organic molecule like methylammonium (CH_3_;NH_3_^+^) or an inorganic ion such as cesium (Cs^+^). ‘B’ is a divalent metal cation, commonly lead (Pb^2+^) or tin (Sn^2+^), while ‘X’ is a halogen anion, such as iodide (I^−^), bromide (Br^−^), or chloride (Cl^−^) [28,29]. Such a configuration forms a three-dimensional framework that is characterized by an arrangement of corner-sharing BX_6_ octahedra, and the ‘A’ cations occupy the interstitial spaces between these octahedra.

The versatility of the perovskite structure allows for significant tuning of electronic and optical properties, a key factor in achieving high efficiency in solar cells [30,31]. This adaptability, coupled with advanced manufacturing techniques such as solution processing, presents a cost-effective alternative to traditional silicon-based solar cells [32]. By allowing the adjustment of the bandgap and having a high light absorption coefficient, perovskites can absorb sunlight efficiently even within thin layers [33]. This property not only enhances their photovoltaic efficiency but also contributes to their flexibility and lightweight characteristics, making them highly suitable for a wide range of applications [34]. Moreover, the ease of tuning perovskite compositions enables optimization for different light absorption spectra, enhancing their suitability for diverse climatic conditions [35]. As a result, perovskite solar cells are positioned as a promising technology that could revolutionize solar energy by offering a high-performance, low-cost, and versatile solution for sustainable energy generation.

However, transitioning PSCs from small laboratory cells to large modules suitable for commercial use presents numerous challenges. Growing highly uniform and crystalline perovskite thin films through solution processing is particularly challenging due to various phenomena during film formation, such as solvent evaporation, wetting effects, inhomogeneous film stress, and uncontrolled nucleation and growth. Understanding the different stages of perovskite crystallization is crucial for producing high-quality films and achieving higher PCEs [36]. As module size increases, maintaining the high efficiency and stability observed in smaller devices becomes increasingly difficult [37]. Issues such as achieving uniform film deposition, preventing defect formation, and mitigating material degradation under environmental stress become more pronounced at larger scales.

Developing scalable fabrication techniques, such as slot-die coating, is essential to ensure consistent quality across large surface areas. Addressing these challenges requires a multidisciplinary approach, integrating advances in materials science, engineering, and process optimization. Successfully overcoming these obstacles could enable PSCs to revolutionize the photovoltaic industry, offering a cost-effective and efficient alternative to existing solar technologies.

## 3. Fundamentals and Advantages of Slit Coating

### 3.1. Slit Coating Method

The slit coating method is a highly promising technique for fabricating large-area PSCs, particularly due to its compatibility with roll-to-roll systems. Notably, perovskite materials not only exhibit excellent optoelectronic properties, but their solution processability also allows devices to be fabricated on substrates through roll-to-roll processes, further enhancing the application potential of the slit coating method [33]. The method involves the precise application of a coating liquid through a narrow slit from a die, allowing for meticulous control over the thickness of the film deposited on a substrate [24,25]. Known for its ability to maintain high uniformity, the slit coating process is versatile enough to accommodate a wide range of liquid viscosities, ensuring the production of high-quality perovskite layers essential for efficient solar cell performance [38].

**Figure 2:**
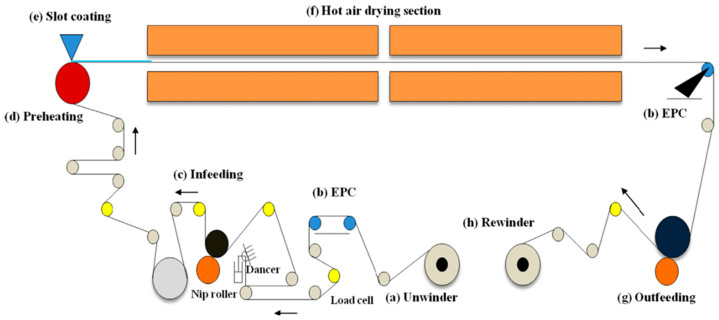
Stable coil conveying process in a roll-to-roll slit die coating system for high coating performance. (**a**) Uncoiler. (**b**) Edge Positioning Controller (Edge Positioning Controller: EPC). (**c**) Feeders. (**d**) Preheating rollers (spare). (**e**) Slit die coaters. (**f**) Hot airdrying section. (**g**) Dispenser. (**h**) Rewinder [40].

The slit coating method, originally invented by Beguin, is a continuous coating process that involves applying an extruded liquid film onto a moving substrate, primarily used for producing photographic film that covers multiple layers of photosensitive materials [22,39]. Its unique ability to directly control film thickness by adjusting the liquid flow rate and substrate speed has led to widespread industrial applications. A typical R2R system, as depicted in Figure 2, includes several key components: an unwinder, edge position controller (EPC), oscillating rollers, feeders, preheaters, hot air dryers, dischargers, and rewinders. The components collectively facilitate a stable coil conveying process, ensuring high coating performance, continuous production, high throughput, low cost, and consistent quality [25,40]. The adaptability of the slit coating method to flexible substrates and large-scale applications makes it particularly advantageous for the cost-effective scaling up of PSC production.

**Figure 3:**
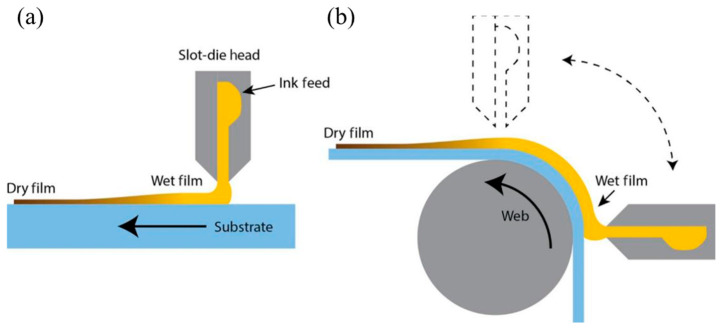
Diagram of the slit coating process: (**a**) sheet-to-sheet, (**b**) roll-to-roll [42].

In 2014, the introduction of pre-metered processes by Vak, which included slit coating, addressed many limitations of existing coating techniques [41]. By precisely regulating the liquid flow rate and substrate speed, they achieved PSCs with power conversion efficiencies exceeding 6%. This achievement spurred significant research and development in slit coating technology, enhancing its scalability and efficiency in solar cell fabrication. Figure 3 illustrates the versatility of the slit coating method, showcasing both sheet-to-sheet and roll-to-roll configurations. These formats highlight the method’s adaptability to different production scales, further underscoring its role in advancing the commercial viability of perovskite solar cells [42].

### 3.2. Advantages of Slit Coating for Large-Area Perovskites

The development of slit coating methods for PSCs has been driven by the need to improve PCE and scalability. Various deposition techniques have been explored, each with its own set of advantages and disadvantages. Below is a summary of these methods, as illustrated in Table 1, highlighting the benefits and limitations of slit coating in comparison to other techniques.

Based on the comparison, the slit coating method offers distinct advantages for the fabrication of large-area perovskite solar cells, making it a superior choice over traditional approaches like spin coating and evaporation techniques. As a precision coating method, slit coating operates by injecting a perovskite precursor into a slit mold, where the precursor liquid is extruded and spreads across the substrate surface under controlled pressure and flow conditions [54]. This process effectively controls the thickness of the film, a critical factor in high-performance solar cells. The adjustable width and distance of the slit ensure precise control over the coating quality, making it possible to form a homogeneous and high-quality film. Furthermore, the slit coating method is faster than conventional silicon photovoltaic processes, presenting opportunities for deploying new modules with greater efficiency and scalability.

The slit coating method demonstrates significant material efficiency and throughput advantages. It excels in minimizing precursor waste compared to other deposition techniques such as spin coating or flash evaporation, leading to reduced material costs and environmental impact [55]. The method supports rapid deposition over large areas, aligning well with industrial scalability requirements while maintaining precise control over the preparation area and reducing losses. Additionally, it is highly adaptable to flexible substrates, expanding its applicability to diverse solar cell configurations. In summary, the slit coating method offers: (1) higher material utilization; (2) precisely controlled film thickness; (3) compatibility with large-area applications; and (4) adaptability to flexible substrates, making it an ideal choice for advanced perovskite solar cell production.

## 4. Key Advancements in Slit Coating for Perovskites

In recent years, slit coating technology has made significant progress in the manufacturing of PSCs. It has not only excelled in improving production efficiency but has also demonstrated great potential in enhancing device performance and the feasibility of large-scale commercial production. The following sections will explore key technological breakthroughs in slit coating for PSCs, focusing on innovative processes, advanced deposition techniques, additive technology, and innovations in transport layers.

### 4.1. Solvent Engineering and Perovskite Deposition Processes

**Figure 4:**
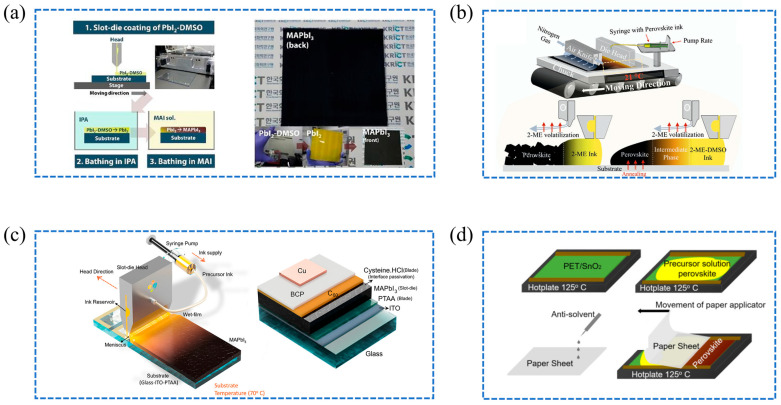
(**a**) Illustration of large-area perovskite thin film preparation using slot-die coating; a photograph of a 10 × 10 cm^2^ perovskite thin film produced by this method. Inset shows images of PbI_2_-DMSO, PbI_2_, and MAPbI_3_ films [56]. (**b**) Schematic of the slot-die coater setup, detailing the coating processes involving 2-ME and 2-ME-DMSO inks [57]. (**c**) The slot-die coating technique for one-step MAPbI_3_ deposition on ITO-PTAA substrates at 70 °C, along with the planar p-i-n MAPbI_3_ perovskite solar cell architecture [58]. (**d**) Schematic illustration of perovskite film deposition using an antisolvent soaked applicator method [59].

In the development of perovskite solar cells, solvent engineering has emerged as a critical factor in optimizing film quality and device performance. Young Yun Kim et al. highlighted the critical role of solution-based methods in their mediator extraction treatment, a fast two-step process that efficiently converts a PbI_2_-DMSO complex into high-quality MAPbI_3_ films [56]. The solution-based approach enables precise control over crystallization, achieving a power conversion efficiency with slot-die coating on a 10 × 10 cm^2^ substrate. This underscores its effectiveness for fabricating large-area PSCs at room temperature, with the specific steps illustrated in Figure 4a. Eva Unger et al. demonstrated the importance of controlling the DMSO content and the age of 2-methoxy-ethanol-based (2-ME) precursor inks to achieve high-efficiency slot-die coated MAPbI_3_ perovskite solar cells, reaching efficiencies as high as 20.8% [57]. Figure 4b shows a schematic of the slot-die coater setup, detailing the coating processes involving 2-ME and 2-ME-DMSO inks. Maintaining optimal DMSO levels is crucial to prevent the formation of unwanted intermediate phases and to promote the desired perovskite phase, thereby enhancing the morphology of thin films and overall device performance.

Strategic solvent mixing has also played a significant role in the enhancement of perovskite thin films. The strategic mixing of low boiling point (BP) solvents like 2-methoxyethanol (2-ME) and acetonitrile (ACN) with high BP solvents such as DMF and DMSO has significantly enhanced the quality of perovskite thin films. Deng et al. demonstrated improved film uniformity and densification using an ACN/2-ME system, achieving PCEs of 21.3% and 16.9% for different cell areas [60]. Subbiah et al. developed a mixed MAPbI_3_ ink solution with ACN and methylamine in methanol (6:4 *v*/*v*) to enhance perovskite dissolution. Through slot-die coating at 70 °C and post-annealing at 100 °C for 10 min, they achieved a PCE of 18.1% for 0.1 cm^2^ areas [58]. The approach is illustrated in Figure 4c, which details the slot-die coating technique and its key parameters for one-step deposition of MAPbI_3_ on ITO-PTAA substrates. The researchers highlight the potential of solvent engineering to tailor the film deposition process, promoting high-quality film formation while enabling scalable PSC manufacturing processes.

Perovskite deposition processes also play a crucial role in determining the efficiency and scalability of perovskite solar cells. According to Tu et al., perovskite deposition processes can be categorized into one-step and two-step methods [25]. In the one-step deposition process, a precursor solution is directly applied to a substrate, followed by annealing and drying at an appropriate temperature to form a perovskite film. The one-step deposition method, while straightforward and cost-effective, often results in lower surface coverage of perovskites. Conversely, the two-step method involves separately applying the inorganic and organic components, which is better suited for precision but less ideal for large-scale production due to its complexity. Selection between these methods should be tailored to the specific context and application needs. Pioneering work by Hwang et al. demonstrated the feasibility of the two-step slit coating method in preparing PSCs, achieving a PCE of 11.96% over an area of 1 × 1 cm^2^ under environmental conditions [8]. Such achievement underscores the practical application of the two-step deposition in PSC fabrication. In exploring commercial-scale slit coating for large-area perovskites, Kim et al. identified challenges posed by the absence of an inherent drying mechanism in slit coating compared to spin coating [61]. To address this challenge, they developed a one-step slit coating method combining gas quenching and substrate heating, resulting in a dense film and achieving a PCE of 12.7% for devices produced in air (10 mm^2^). In another innovative approach, Zarabinia et al. introduced the Anti-Solvent Immersion Applicator (DASSA) deposition technique, which uses a simple piece of paper as an applicator to deposit uniform, high-quality perovskite films at low temperatures, as illustrated in Figure 4d [59]. This novel method achieved a PCE of 11% for flexible devices over an area of 6 × 4 cm^2^. The DASSA technique is particularly advantageous because it does not require expensive equipment and holds the potential for full automation, opening opportunities for further optimization and future development in large-scale, cost-effective perovskite solar cell manufacturing.

### 4.2. Equipment Innovations and Process Enhancements

**Figure 5:**
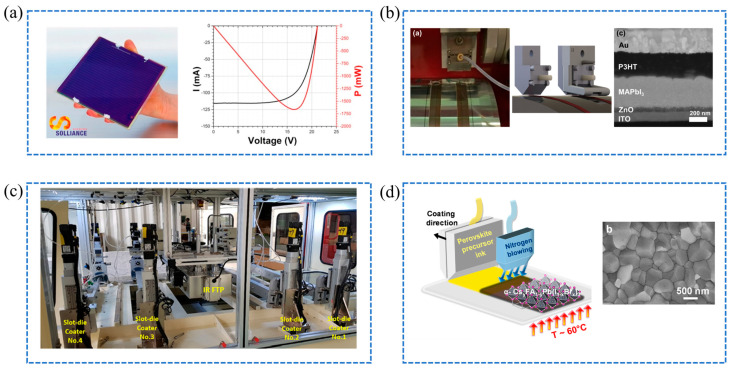
(**a**) A 6 in. × 6 in. perovskite module with an actual dimension of 168.75 cm^2^, comprising 25 interconnected cells, along with its IV and power curves [62]; (**b**) The slot-die coating process for the perovskite absorber, a drawing of the slot-die head with and without an air flow nozzle for drying, and an SEM cross-section of a coated perovskite solar cell on a flexible PET substrate [63]; (**c**) The overall configuration of MK-20 during the slot-die process [64]; (**d**) Gas quenching and substrate heating for crystallizing slot-die coated precursor films into cubic perovskite, with a top-view SEM image of the perovskite film [65].

In the field of scalable fabrication for perovskite solar cells, several innovative techniques have propelled the efficiency and practicality of these devices. Francesco et al. pioneered the chip-to-chip process, a scalable fabrication technique that effectively transitions from small-scale testing to larger 6-inch by 6-inch modules, as depicted in Figure 5a. This method achieves module efficiencies exceeding 10% with minimal efficiency loss compared to traditional spin coating [62]. Mikas et al. enhanced substrate surface treatment by replacing ozone processing with corona technology, compatible with roll-to-roll processes [63]. As demonstrated in the preparation steps shown in Figure 5b, this advancement drastically reduced PbI_2_ treatment times from 15 min to 0.1 s, significantly boosting manufacturing speed while maintaining film quality.

Further advancements focus on equipment and process innovations to enhance perovskite solar cell production. Huang et al. introduced the MK-20, an innovative, low-cost device for manufacturing perovskite solar cells. The configuration of the MK-20, as depicted in Figure 5c, highlights its advanced design and key features, supporting efficient large-scale production. This machine enables continuous heating across four film layers, achieving film fabrication efficiencies of up to 14.3% for 80 cm × 80 cm substrates and facilitating the production of up to six devices per hour [64]. Their work also combined slit coating with near-infrared annealing and nitrogen-free techniques, reaching a PCE of 12.3% for planar inverted PSCs. Zhao et al. demonstrated that higher annealing temperatures improve perovskite crystal growth, where precise control of the feed rate is essential for uniform film formation [66].

In the pursuit of superior film quality, several researchers have made significant contributions through innovative methods and materials. Fievez et al. employed a co-crystallization method combined with gas quenching and substrate heating, as illustrated in the schematic shown in Figure 5d, achieving an impressive efficiency of 18% on a 0.09 cm^2^ substrate [65]. This approach highlights the importance of controlling crystallization dynamics and thermal management to enhance film uniformity and device performance. Meanwhile, Lao et al. introduced a dual-blade slot die method, significantly improving the precision of precursor application and resulting in an impressive efficiency of 19.7% for 9 mm^2^ perovskite devices [67]. By utilizing dual blades, the technique ensures a more uniform distribution of the perovskite precursor, reducing the need for high precursor concentrations and enhancing device reproducibility. This advancement provides a scalable solution for producing high-quality perovskite thin films, making it suitable for industrial applications.

Continuing with advancements in processing techniques, Ciro et al. explored rapid solution processing for high-performance p-i-n structures, achieving a device efficiency of 2.9% on a 12 mm^2^ substrate [68]. Although the initial efficiency is modest, the research opens promising new directions for PSCs development. By optimizing deposition parameters and utilizing a mini-coating machine, Ciro et al. demonstrated precise control over the properties, thickness, and roughness of device layers under various environmental conditions. This capability facilitates the adoption of low-temperature processes on flexible substrates, which can significantly reduce production costs and expand the applicability of PSCs. Daniel et al. demonstrated the effectiveness of the slit coating method in achieving a stabilized PCE of 7% with PSCs using four-layer slit coating films over a 5 × 5 cm^2^ area, proving superior to traditional spin coating techniques [69]. Finally, Cotella et al. successfully employed a two-step process for one-step deposition on mesoporous TiO_2_ scaffolds, achieving an efficiency of 9.2% with a 10 × 15 cm^2^ device [70]. This innovative approach underscores the potential of slit coating for the deposition of halide perovskite layers in one-step processes, further enhancing the scalability and efficiency of perovskite solar cell production. These advancements collectively highlight the diverse strategies being employed to improve film quality and device performance, paving the way for the efficient and scalable production of perovskite solar cells capable of meeting industrial demands.

### 4.3. Enhancing Device Performance Through Additive Technology

To elevate the quality and performance of perovskite films prepared by slit coating, researchers have extensively explored various additives. The additives primarily improve film quality through two mechanisms: by chemically interacting during formation to enhance structural properties, and by passivating defects to increase stability and performance.

**Figure 6:**
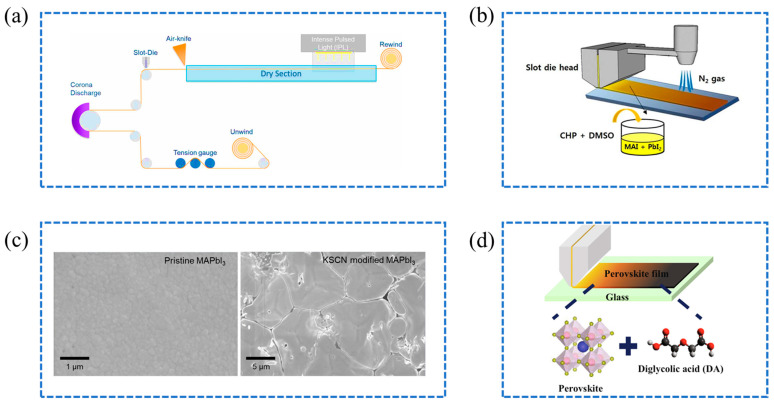
(**a**) Schematic of a roll-to-roll machine setup, detailing continuous processing from surface preparation to thermal processing [71]; (**b**) Schematic of the CHP-DMSO modified slot-die coating process for depositing the perovskite layer [72]; (**c**) SEM of both pristine and KSCN-modified MAPbI_3_ films [73]; (**d**) Schematic of slot-die-coating using DA as an additive for the perovskite film [74].

Additives play a crucial role in enhancing the chemical interactions during perovskite film formation, leading to higher-quality films. Chandrasekhar et al. demonstrated a rapid and scalable roll-to-roll fabrication process for flexible perovskite solar cells, which integrates slot-die coating with intense pulsed light annealing, achieving a power conversion efficiency of 11.24% for a 1 cm^2^ device [71]. The combined use of L-α-phosphatidylcholine (LP) surfactant and DMSO is crucial in enhancing film quality and uniformity, as shown in Figure 6a, thereby boosting the overall efficiency and scalability of the manufacturing process. Jung et al. explored the use of two processing additives, N-cyclohexyl-2-pyrrolidone (CHP) and DMSO, to enhance the quality of MAPbI_3_ films [72], as illustrated in Figure 6b. The additives were shown to facilitate the development of uniform and homogeneous perovskite films in ambient conditions. Specifically, CHP, due to its high boiling point and low vapor pressure, is critical for ensuring film uniformity, while DMSO enhances perovskite crystal growth by creating intermediate states with precursor molecules. As a result, they achieved PSCs with a high PCE of 12.56% and excellent reproducibility. Xu et al. focused on improving the crystal size and quality of slot-die-coated MAPbI_3_ perovskite films through additive engineering with potassium thiocyanate (KSCN) [73]. The approach resulted in micrometer-thick films with an average grain size of approximately 11 μm and charge-carrier parameters comparable to single-crystal perovskites, as shown in the SEM image in Figure 6c. The enhanced properties facilitated the fabrication of planar inverted PSCs with minimal hysteresis, achieving a maximum power conversion efficiency of 21.38%, further underscores the potential of additives in advancing the performance and scalability of slot-die-coated perovskite solar cells. Lee et al. introduced diglycolic acid (DA) as a novel additive in the slot-die-coating process, as depicted in the schematic illustration in Figure 6d, significantly enhancing the efficiency and stability of perovskite solar cells [74]. The DA-based devices achieved a maximum power conversion efficiency of 14.63% and maintained 80% of their initial efficiency over 95 days, further demonstrating the beneficial role of additives in the slot-die-coating process.

**Figure 7:**
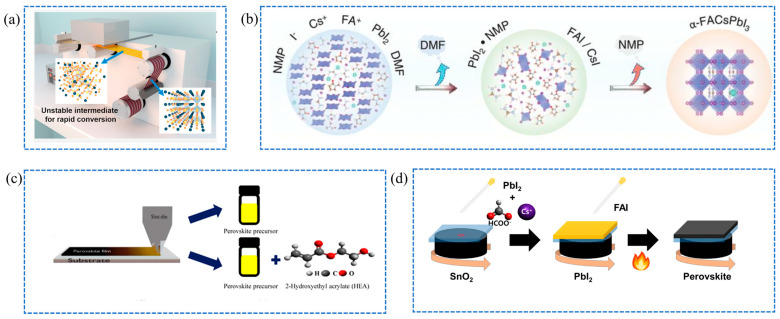
(**a**) Schematic of sequential slot-die coating for the modified perovskite layer [75]. (**b**) Crystal growth with NMP [76]. (**c**) Device architecture and slot-die coating with/without HEA [77]. (**d**) Sequential perovskite deposition with HCOOCs additives [79].

Moreover, additives significantly contribute to the passivation of defects and the improvement of stability in perovskite films by reducing non-radiative recombination and trapping density. Heo et al. developed a printing-friendly sequential deposition technique featuring an intra-additive approach, as illustrated in Figure 7a, utilizing Methylammonium Iodide (MAI) as an internal additive [75]. This modified method led to fully slot die-coated perovskite solar cells achieving a power conversion efficiency of 14.4% under ambient conditions, while roll-to-roll processed flexible cells exhibited an efficiency of 11%. Bu et al. developed a lead halide-templated crystallization strategy using a stable PbI_2_·N-methylpyrrolidone (NMP) adduct to prevent solvent-coordinated intermediate complexes [76], as illustrated in the schematic mechanism shown in Figure 7b. The adduct-assisted approach resulted in high-performance, hysteresis-free perovskite solar cells, with slot die-printed minimodules achieving efficiencies of 20.42% on 17.1 cm^2^ and 19.54% on 65.0 cm^2^. Lee et al. introduced 2-hydroxyethyl acrylate (HEA) as a functional additive to enhance the quality of slot-die-processed perovskite films by improving crystal growth (Figure 7c), enlarging grain size, and reducing defects [77]. This approach resulted in perovskite solar cells with a high efficiency of 16.08% and good stability. Zang et al. introduced N-methyl-2-piperidone as a green additive, to facilitate the one-step production of large-area films, achieving a remarkable efficiency of 20.98% over a 25 cm^2^ area while promoting safer operational practices [78]. Baek et al. introduced cesium formate as a chemical additive in perovskite solar cells to passivate defects and improve roll-to-roll processability, resulting in enhanced crystallinity and reduced defect density [79]. The approach demonstrated improved stability and efficiency retention in air, achieving efficiencies exceeding 14% on 2.5 × 2.5 cm^2^ areas. In conclusion, the advancements underscore the critical role of additives in enhancing the performance and durability of perovskite solar cells, paving the way for more efficient and sustainable photovoltaic technologies.

### 4.4. Innovations in Electron and Hole Transport Layers

High-quality electron transport layer (ETL) and hole transport layer (HTL) are crucial for achieving efficient PSCs, as they facilitate effective charge extraction and transport. In recent years, significant progress has been made in large-scale fabrication techniques for ETLs and HTLs, including chemical vapor deposition (CVD) [80,81], Pulsed laser deposition (PLD) [82], and other R2R methods [83]. Among various preparation techniques, slot-die coating stands out for its precise control over film thickness and quality through adjustable slit parameters, enabling the formation of homogeneous, high-performance ETLs and HTLs crucial for efficient, large-area PSCs.

**Figure 8:**
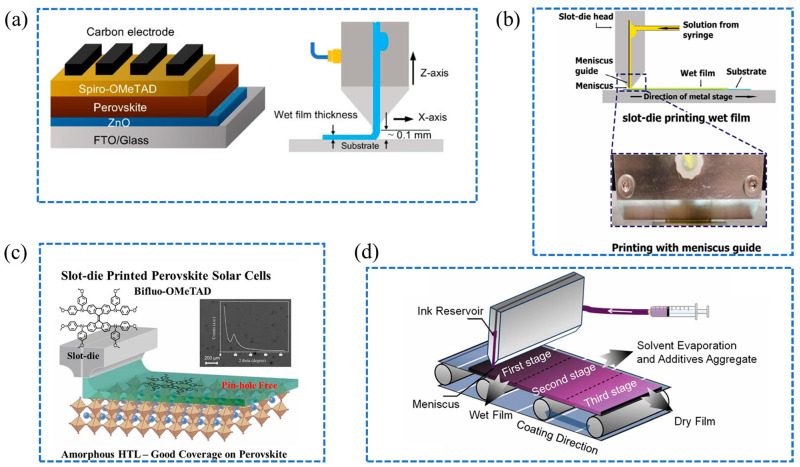
(**a**) Schematic of ZnO-based device structure and slot-die coating process [84]; (**b**) Film preparation process of the slot-die coater [85]; (**c**) Schematic diagram of the slot-die printed perovskite solar cell with Bifluo-OMeTAD [86]; (**d**) The slot-die coating process for PSCs.

Traditionally, in conventional planar solar cell architectures, titanium dioxide (TiO_2_) and Spiro-OMeTAD are employed as the ETL and HTL, respectively. However, fabricating TiO_2_ for effective electron transport in the ETL often requires high processing temperatures (above 400 °C), which pose challenges for large-scale production and limit the use on flexible substrates. As an alternative, zinc oxide (ZnO) offers enhanced optoelectronic properties and superior electron mobility. Khambunkoed et al. developed a method for producing fully-covered ZnO thin films as ETLs using slot-die coating, achieving a PCE of 10.81% in carbon-based PSCs [84]. The ZnO-based device structure and the slot-die coating process are shown in Figure 8a. The improvement in ETL quality resulted in reduced interfacial charge recombination and enhanced reproducibility and storage stability over 500 h, demonstrating the potential for scalable and cost-effective thin film deposition. Le et al. demonstrate that in slot-die-coated inverted PSCs, ETLs such as PCBM and BCP achieve improved morphology through vacuum quenching (Figure 8b), contributing to power conversion efficiencies exceeding 16% for MAPbI_3_-based devices and over 17% for CsFAPbI_3_-based devices [85].

In parallel with these developments in ETL materials, innovations in HTL materials have also been significant. Qin et al. demonstrated the use of Bifluo-OMeTAD as an amorphous HTL in fully slot-die coated perovskite solar cells, achieving a power conversion efficiency of 14.7% [86]. The molecular design of Bifluo-OMeTAD effectively suppresses crystallization, enhancing film formation and offering a promising alternative to Spiro-OMeTAD for scalable roll-to-roll production of perovskite solar cells. Yin et al. emphasize the critical importance of high-quality, large-area HTLs in preserving PCE in scalable PSCs [87]. Using slot-die coating and optimized solvent engineering, uniform, pinhole-free HTL films are achieved (Figure 8d), enhancing hole extraction and minimizing interfacial recombination, resulting in an average efficiency of 17.7% and 85% performance retention after 1000 h of aging. Du et al. introduced a surface redox engineering (SRE) technique for electron-beam evaporated NiO_x_, enhancing its compatibility as an HTL in slot-die-coated large-area PSCs [88]. The approach not only resolves de-wetting issues with slot-die-coated perovskite films but also enhances electronic properties at buried interfaces. The resulting PSCs exhibited excellent stability, with a 156 × 156 mm^2^ module achieving an impressive efficiency of 18.6%. The advances in electron and hole transport layer technologies demonstrate significant potential for enhancing PSC performance, facilitating better scalability, and paving the way for innovative production techniques.

## 5. Challenges and Future Prospects

### 5.1. General Challenges

The application of slot coating in the fabrication of PSCs presents both challenges and opportunities. While slot-die coated PSCs have achieved efficiencies exceeding 21%, rivaling those produced by other fabrication methods, the power conversion efficiency often remains lower than the record-setting devices created using spin coating. This discrepancy is largely attributed to the relatively limited research dedicated to optimizing perovskite deposition specifically through the slot coating method.

Innovative Processes. One of the primary challenges lies in the nucleation and crystallization processes during film formation. By improving innovative methods such as adjusting substrate temperature, liquid level, and coating head speed, it is expected to achieve a more uniform and defect-free film, thereby enhancing equipment performance. Furthermore, precise control over these parameters can lead to the formation of optimal crystal orientations and grain boundaries, which are crucial for maximizing charge carrier mobility and minimizing recombination losses.

Advanced Deposition Approaches. Refining the composition and stability of precursor solutions is crucial for producing high-quality perovskite films, especially in slot-die coating processes. Ensuring uniformity and stability in these solutions leads to consistent film formation, minimizing defects and boosting the large area solar cell efficiency. Furthermore, the precise tuning of solvent systems and additives can control the crystallization kinetics and film morphology, enabling the formation of dense, pinhole-free layers with optimal grain size and orientation, which are essential for enhancing charge transport and reducing recombination losses in large-area devices.

Device Structure Optimization. The optimization involves the meticulous selection of materials with high charge carrier mobility and suitable energy levels to ensure efficient electron and hole transport. Additionally, precise engineering of the electronic band alignment between layers is crucial for optimal conduction and valence band offsets, facilitating seamless charge transfer and minimizing recombination losses. Effective passivation of interfacial defects further enhances device performance by reducing non-radiative recombination. Moreover, engineering the thickness and morphology of transport layers can improve charge transport and reduce resistance.

### 5.2. Stability Challenges in PSCs

Addressing the general challenges, as discussed in the previous section, is essential for achieving high-performance PSCs. However, stability stands out as a particularly crucial aspect that demands further exploration. The performance and durability of PSCs are significantly influenced by the stability of its various components.

Photoactive Layer Stability. The stability of the photoactive perovskite layer is compromised by exposure to moisture, oxygen, and light, which trigger hydrolysis, oxidation, and photo-degradation processes, respectively, ultimately leading to deterioration in performance and efficiency. Recent research has focused on multiple strategies to enhance the stability of perovskite photoactive layers. These include compositional engineering through the incorporation of elements like cesium or rubidium, structural modifications such as 2D/3D hybrid architectures, and advanced encapsulation methods designed to protect the layer from environmental degradation, collectively aiming to improve long-term device performance and durability.

Interfaces with Transport Layers. The stability of PSCs is significantly influenced by the interfaces between the transport layers (HTL and ETL) and the perovskite layer. Chemical incompatibilities at these interfaces can lead to ion migration, unwanted reactions, and defect formation, all of which can compromise device performance. For the HTL-perovskite interface, strategies like using hydrophobic HTLs, introducing buffer layers, and employing dopant-free HTLs are employed to mitigate chemical reactions and reduce ionic movement, which can be effective in improving stability of PSCs. At the ETL-perovskite interface, surface passivation techniques, integrating metal oxide nanoparticles for enhanced electron extraction, and developing low-temperature processable ETLs compatible with flexible substrates are implemented to enhance stability. These innovations aim to minimize defects, improve charge transport, and ultimately enhance the overall stability and efficiency of PSCs.

### 5.3. Future Directions for Slot-Die Coated PSCs

Optimizing Slot-Die Coating. To improve the performance of slot-die coated PSCs, more research is needed on optimizing perovskite deposition through slot-die coating. This includes further investigations into improving the nucleation and crystallization processes, optimizing the composition and stability of precursor solutions, and engineering the device structure for better performance.

Scalability and Commercial Viability. The transition from laboratory-scale to commercial-scale production presents challenges. Maintaining high efficiency and stability across large surface areas is essential for commercial viability. This requires addressing issues such as achieving uniform film deposition, preventing defect formation, and mitigating material degradation under environmental stress. Continued development of scalable fabrication techniques like slot-die coating is necessary, along with efforts to make the production process more efficient and cost-effective.

Integrating Stability Strategies. Given the importance of stability, future research should focus on integrating the stability enhancement strategies into scalable manufacturing processes. This will ensure that the benefits of slot-die coating can be fully realized in commercial PSC production.

## Figures and Tables

**Table 1:** Advantages and disadvantages of preparation methods for large-area perovskites.

Methods	Advantages	Shortcomings
Spin coating method [43]	Simple preparation, Controllable adjustment, Stable performance in small area	Incompatible with mass production, serious waste, sharp decline in large-area performance
Spraying method [44]	Low cost, less waste, can be mass-produced	Poor performance
Blade coating method [45]	Simple and easy to prepare, strong adjustable ability, high efficiency	Uneven quality, difficult to balance
Inkjet printing [46]	Direct modeling, effect pulling group, extensible, to achieve special functions	Difficulties in film formation, Serious waste
Vacuum flash assisted method [47]	Excellent film, industrial availability, non-toxic environmental protection, controllable film thickness, low temperature requirements	Low carrier transport, Poor stability
Chemical vapor deposition [48]	Large area application, uniform film, high repeatability, commercial prospects	Amorphous film, defective crystal, poor charge carrier transport, high cost
Sequential evaporation method [49]	Excellent small area performance, compact film, good thickness control, high repeatability, good quality	Unfavorable commercial, high cost
Co-evaporation method [50]	Smooth and uniform film, good film effect, no annealing, compatible with flat battery	High cost, difficult to control
Flash evaporation [47]	Highly uniform, high surface roughness, extensibility, additive, very fast	High cost, difficult to fabricate
Vacuum thermal evaporation [51]	Simple wide application, scalability, industrial preparation, control film	Waste is serious, cost is high, additives are difficult to add
Multi-flow air knife method [52]	Suitable for large area PSC, good replication, low cost	Requires careful control of the space between the air knife and the solution film
Drip casting method [53]	High material utilization, can control film thickness, fast and cheap	Uneven evaporation, difficult to control rate

## Data Availability

Not applicable.
